# Assessing Spectral Analysis of Phytoconstituents and Their *In Silico* Interactions with Target Proteins in Plant Seed Extracts

**DOI:** 10.3390/plants12193352

**Published:** 2023-09-22

**Authors:** Venkatadri Babu, J Irshad Ahamed, Agastian Paul, Sajad Ali, Irfan A. Rather, Jamal S. M. Sabir

**Affiliations:** 1 Department of Plant Biology and Biotechnology, Loyola College (Autonomous), Affiliated to University of Madras, Chennai 600034, Tamil Nadu, India; 2 Department of Chemistry, Loyola College (Autonomous), Affiliated to University of Madras, Chennai 600034, Tamil Nadu, India; 3 Department of Biotechnology, Yeungnam University, Gyeongsan-si 385541, Gyeongbuk, Republic of Korea; 4 Department of Biological Sciences, Faculty of Science, King Abdulaziz University (KAU), Jeddah 21589, Saudi Arabia; 5 Centre of Excellence in Bionanoscience Research, King Abdulaziz University (KAU), Jeddah 21589, Saudi Arabia

**Keywords:** vegetable seed, GC-MS, antibacterial, antioxidant, anti-breast cancer, in-silico molecular docking

## Abstract

The pharmacological and preventive attributes of extracts from vegetable seeds have garnered widespread recognition within the scientific community. This study systematically assessed the in vitro antibacterial, antioxidant, and anti-breast cancer properties of phytochemicals present in various solvent-based vegetable seed extracts. We also conducted molecular docking simulations to ascertain their interactions with specific target proteins. Besides, nine distinct chemical constituents were identified using gas chromatography-mass spectrometry (GCMS). Remarkably, the ethyl acetate extract exhibited robust inhibitory effects against Gram-positive and Gram-negative bacterial strains. Furthermore, its capacity for 2,2-diphenyl-1-picrylhydrazyl (DPPH) scavenging was found to be noteworthy, with an IC_50_ value of 550.82 ± 1.7 µg/mL, representing a scavenging efficiency of 64.1 ± 2.8%. Additionally, the ethyl acetate extract demonstrated significant hydrogen peroxide (H_2_O_2_) scavenging activity, with a maximal scavenging rate of 44.1 ± 1.70% (IC_50_) at a concentration of 761.17 ± 1.8 µg/mL. Intriguingly, in vitro cytotoxicity assays against human breast cancer (MCF-7) cells revealed varying levels of cell viability at different extract concentrations, suggesting potential anticancer properties. Importantly, these ethyl acetate extracts did not display toxicity to L929 cells across the concentration range tested. Subsequently, we conducted in-silico molecular docking experiments utilizing Discovery Studio 4.0 against the c-Met kinase protein (hepatocyte growth factor; PDB ID: 1N0W). Among the various compounds assessed, 3,4-Dihydroxy-1,6-bis-(3-methoxy-phenyl)-hexa-2,4-diene-1,6-dione exhibited a notable binding energy of −9.1 kcal/mol, warranting further investigation into its potential anticancer properties, clinical applications, and broader pharmacological characteristics.

## 1. Introduction

The prevalence of cancer worldwide is staggering, with 277 distinct varieties identified to date. This devastating disease often has hereditary implications, compounding its impact on individuals and families. Furthermore, it was reported that by 2040, there will be 28.4 million new cancer cases worldwide [1]. The two leading causes of death in people worldwide are cancer and microbial infections. Infection is the second most frequent cause of death for cancer patients [2]. Infections may be more severe and potentially fatal in cancer patients than in the general population due to their increased immunosuppression prevalence. Environmental factors and chemicals are also one of the major causes of cancer in humans. Our environment houses an astounding range of over 100,000 diverse chemical types. Unfortunately, only a fraction of these chemicals, approximately 35,000, have undergone comprehensive scientific scrutiny. Alarmingly, among the studied chemicals, about 300 have been confirmed as carcinogenic, posing significant health risks. Moreover, an estimated 65,000 natural compounds remain largely unexplored within our natural surroundings, presenting an immense reservoir of potential therapeutic agents and valuable insights yet to be discovered. Investigating these compounds holds enormous promise for advancing our understanding of cancer and exploring novel avenues for prevention and treatment [3,4,5]. 

Uncontrolled cell division, arising from genetic abnormalities and environmental conditions, underlines cancer development. The typical four genes involved in the behavior of cancer cells are DNA repair genes, tumor suppressor genes, oncogenes, and programmed death genes [4,6]. When a genetic mutation occurs in a cell, normal regulatory mechanisms are disrupted, allowing the cell to receive new instructions that lead to its transformation into a cancerous cell. In addition to genetic factors, various environmental agents such as chemicals, ultraviolet radiation, viruses, and bacteria contribute to the initiation and progression of cancer [7,8]. Cancer and other deadly illnesses have existed since the dawn of time.

In the present era, advancements in computational techniques and molecular medicine have provided unprecedented opportunities to investigate the causes and mechanisms of this lethal disease, leading to improved early detection and therapeutic interventions [9]. Over half of all cancer cases are being effectively treated, especially when diagnosed early. Treatment options encompass a range of modalities, including surgery, chemotherapy, radiation therapy, immunotherapy, gene therapy, and combinations [10]. However, the substantial side effects associated with contemporary chemotherapy necessitate the development of more refined treatment approaches. These side effects may include oral ulcers, weight loss, diarrhea, vomiting, hair loss, exhaustion, and nausea [11].

According to data from the International Organization for Research on Cancer for 2020, breast cancer has surpassed lung cancer as the most common disease diagnosed globally for the first time [12]. Nowadays, non-specific cytotoxic medicines comprise most of the chemotherapeutic medications utilized in clinical settings. While these medications destroy tumor cells, they also cause various degrees of harm to other cells and organs [13,14,15]. It is critical to look into the most potent anti-breast cancer medications because of the high postoperative recurrence rate, mortality rate, and metastasis rate, among other factors [16]. The use of traditional ethnic remedies in treating breast cancer is widespread worldwide, and a wealth of knowledge has been amassed in this area [17]. Moreover, different routes and targets have been employed to mitigate the side effects of chemotherapy, including tissue damage, while simultaneously aiming to prevent metastasis and recurrence.

The incidence of breast cancer, the primary cause of mortality in women, is a result of global lifestyle changes [18]. According to the World Health Organization (WHO), about 2.26 million new breast cancer cases were identified globally in 2020, overtaking lung cancer as the most frequently diagnosed malignancy [19]. Surgical excision is the main form of treatment for breast cancer in its early stages, and depending on the patient’s circumstances, adjuvant therapy may then be chosen. Adjuvant treatments include postoperative adjuvant chemotherapy, adjuvant radiation, adjuvant endocrine therapy, and molecular targeted therapy [20]. Molecular targeted therapy has become one of the main treatment techniques for breast cancer because of its small medication dose, high specificity, minimal toxicity, and excellent therapeutic efficacy [21,22].

Since ancient times, medicinal phytoconstituents derived from plants and vegetable seeds have been used to treat various ailments. Most pharmaceuticals today originated in plants, directly or indirectly, and plant-based medications have proved essential for treating a variety of disorders. The global crisis has further propelled the popularity of herbal medicine as a supplemental and alternative therapy [23]. Thus, there is a considerable demand for new alternative medications and finding natural compounds that target many signaling pathways, suppress the proliferation of cancer cells, and have minimal adverse effects on normal cells [24]. In many nations and ethnic groups worldwide, phytomedicines have gained popularity over the past 40 years and are frequently utilized as supplemental or alternative therapies [25]. Due to their safety, efficacy, purity, and accessibility, phytoconstituents extracted from plant and vegetable seeds play a significant role in basic healthcare as natural treatments in most nations [26,27]. According to reports, natural goods or naturally modified products made up 49% of the 175 small molecules authorized for cancer treatment. The presence of 4-(1H)-quinolones and 1,2,3,4-tetrahydroacridin9(10H)-ones (THA) in many natural sources has attracted the attention of organic and pharmaceutical chemists for many years [15,16,17].

Acridine is an anthracene alkaloid in terms of chemistry [18,19]. According to estimates, about 80% of patients cure inflammatory disorders with plant and vegetable seed medications in this situation [28,29]. Vegetable and plant seeds, with their extensive ethnomedicinal history, represent a rich source of bioactive substances that offer therapeutic and health benefits against various ailments [30,31]. Consequently, scientists in the field of ethnoscience are actively working to elucidate the potential adverse effects, determine appropriate dosages, and optimize extraction methods for identifying bioactive components from plants and vegetable seeds. Bioactive substances are secondary plant metabolites with pharmacological or toxicological effects in humans and animals. However, a significant challenge researchers face is that a single plant or vegetable seed may contain multiple bioactive substances [32]. Despite the decline in popularity of this strategy during the 20th century, there is currently a global resurgence of interest in medicinal plants and vegetable seeds as valuable natural goods that could produce the next generation of semi-synthetic derivatives [33].

Computer-driven tools for drug discovery have made it easier to screen potent compounds for medications derived from phytochemicals [34,35]. They have been used for the in silico prediction of pharmacological, pharmacokinetic, and toxicological performance of different phytochemicals [36]. Computational prediction models are crucial to choosing the best pharmacological and technological research methodology [37,38]. Among these, molecular docking is a productive and economical method for creating and testing drugs [37,38]. This method produces information on drug and receptor interactions that can forecast the direction in which drug candidates will bind to their intended protein. Additionally, this method makes systemic study easier by non-covalently introducing a molecule into the binding site of a target macromolecule, resulting in precise binding at each ligand’s active site. From this perspective, the present investigation underpins the antimicrobial, antioxidant, anti-breast cancer, and molecular docking interaction of phytoactive compounds with target proteins from various vegetable seed extracts.

## 2. Results and Discussion

### 2.1. In Vitro Antimicrobial Test

Humans are very often challenged by different microbial pathogens, which pose a serious threat to their survival. With the emergence of antibiotic resistance, they are becoming more lethal, especially in immunocompromised patients. Therefore, there is a need to identify new drugs that can combat human pathogens without causing any harm to humans. In this regard, we systematically examine the effect of seed extracts against different microbial pathogens, as shown in Table 1. Our findings demonstrated that all bacterial strains examined responded differently to ethyl acetate extracts of vegetable seeds’ antibacterial properties. Compared to hexane and methanol, the ethyl acetate extract obtained from seeds proved to be the most significant inhibitory activity against *Staphylococcus epidermis, Micrococcus luteus, Enterobacter aerogens*, and *Proteus vulgaris*. In this study streptomycin was used as a positive control. The ethyl acetate extract demonstrated substantial bactericidal activity with a maximum zone of inhibition against *S. epidermis* (12.6 ± 0.5 mm) and the lowest zone of inhibition against *P. vulgaris* (9.3 ± 0.5 mm), as shown in Table 1.

This antimicrobial activity can be ascribed to Methyl oleate, Isopropyl stearate, Phe-nol,2,6-bis(1,1-dimethylethyl)-4-(4-hydroxy-3,5-dimethylphenyl) methyl)-(22.82) 3,4-Dihydroxy-1,6-bis-(3-methoxy-phenyl)-hexa-2,4-diene-1,6-dione found in ethyl acetate crude extracts. Antimicrobial substances are known to exist in these phytochemical categories [39]. Hence, these inclusions may explain the activity observed in the extracts. Previous studies have also revealed the antimicrobial activity of different plant seeds against different microbial pathogens [26,40]. Similarly, our study further supports the notion that bioactive compounds of seeds are the best resources for combating antibiotic resistance threats in human pathogens. 

### 2.2. Antioxidant Activities

**Figure 1:**
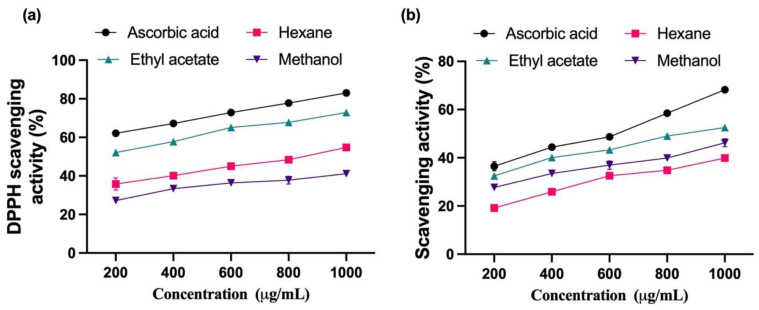
Antioxidant activity of vegetable seed extracts of hexane, ethyl acetate, methanol extracts and ascorbic acid. (**a**) DPPH scavenging effect of different concentrations (200–1000 µg/mL). (**b**) H_2_O_2_ scavenging effect of different concentrations (200–1000 µg/mL) of vegetable seed extracts of hexane, ethyl acetate, methanol extracts, and ascorbic acid. Each value represents the mean ± SEM of triplicate experiments.

In this study, ethyl acetate vegetable seed extracts showed a rise in DPPH degrading abilities in a dose-dependent way. Maximum DPPH scavenging characteristics in functional ethyl acetate seed extracts ranged from 64.1 ± 2.8% (IC_50_) at a 550.82 ± 1.7 µg/mL concentration. The IC_50_ value of ascorbic acid was 450.12 ± 2.25 µg/mL (Figure 1a). Likewise, ethyl acetate vegetable seed extracts showed maximum H_2_O_2_ scavenging trait ranging from 44.1 ± 1.70% (IC_50_) at a 761.17 ± 1.8 µg/mL concentration. The IC_50_ value of ascorbic acid was 650.37 ± 2.15 µg/mL (Figure 1b). Based on the findings, it was found that ethyl acetate seed extracts had more antioxidant activities than hexane and methanol seed extracts. Similar results were also reported in other plant extracts [41].

### 2.3. GC–MS Analysis of Ethyl Acetate Seed Extracts

**Figure 2:**
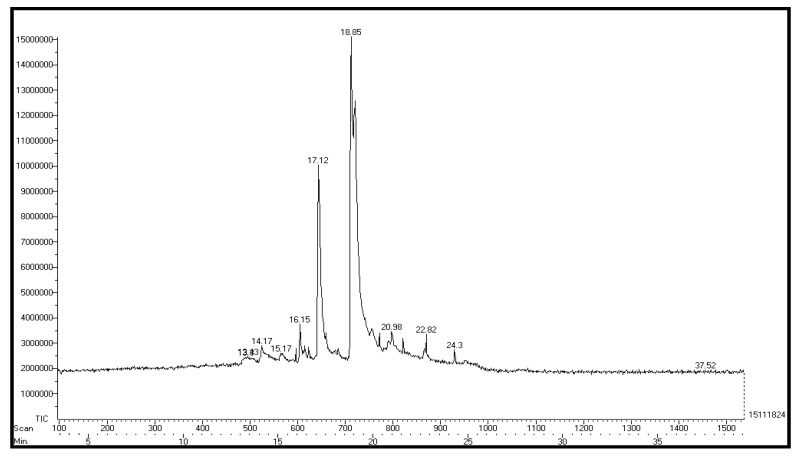
Gas chromatography–mass spectrometry chromatograms of the ethyl acetate extract of vegetable seed (Synergistic).

**Figure 3:**
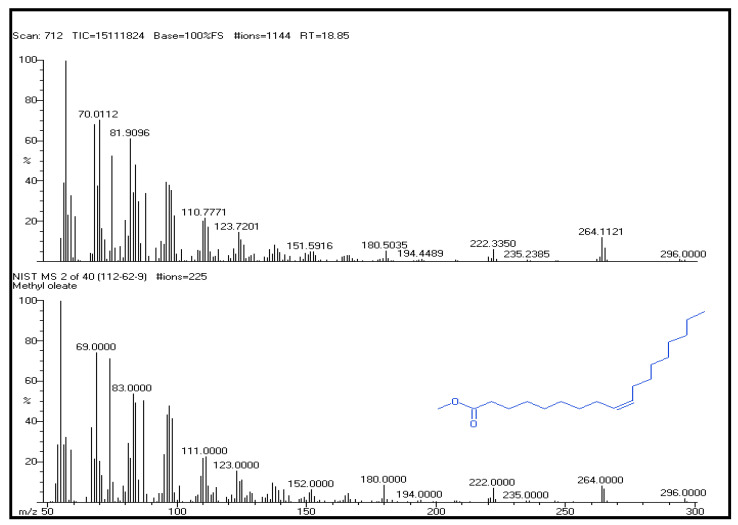
Mass spectrum for the major constituent methyl oleate from the ethyl acetate extract of vegetable seed (Synergistic).

In this study, GC-MS analysis of seed extracts was performed to identify different bioactive compounds that can possess biological activity. Based on the analysis, the GC-MS chromatogram of the seed extracts made from the ethyl acetate is shown in Figure 2. The study found nine bioactive chemicals in the ethyl acetate vegetable seed extracts, as shown in Table 2, along with their relative abundance (area), GC retention time, and compound names. In the GC-MS profile, methyl oleate was the most significant compound, with an 18.85 peak retention time value. In comparison with other compounds, isopropyl stearate (20.98), 2,6-bis(1,1-dimethyl ethyl)-4-(4-hydroxy-3,5-dimethyl phenyl) methyl)-(22.82) 3,4-dihydroxy-1,6-bis-(3-methoxy-phenyl)-hexa-2,4-diene-1,6-dione (24.3) were found in the seed extracts of ethyl acetate varieties. Figure 3 shows the structures of these predominant compounds.

### 2.4. In Vitro Cytotoxicity

**Figure 4:**
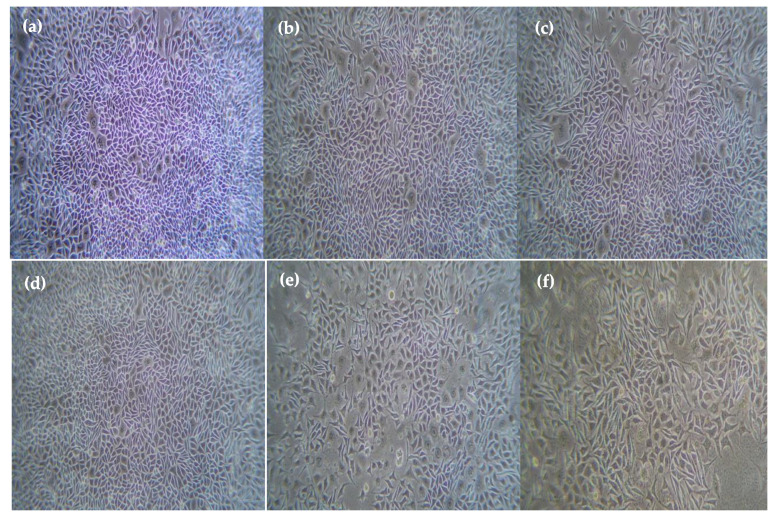
Anticancer activity of L-929 (Fibroblast cells) ethyl acetate extracts (**a**) control, (**b**) 500 µg/ mL, (**c**) 250 µg/mL, (**d**) 100 µg/mL, (**e**) 50 µg/mL, and (**f**) 25 µg/mL.

**Figure 5:**
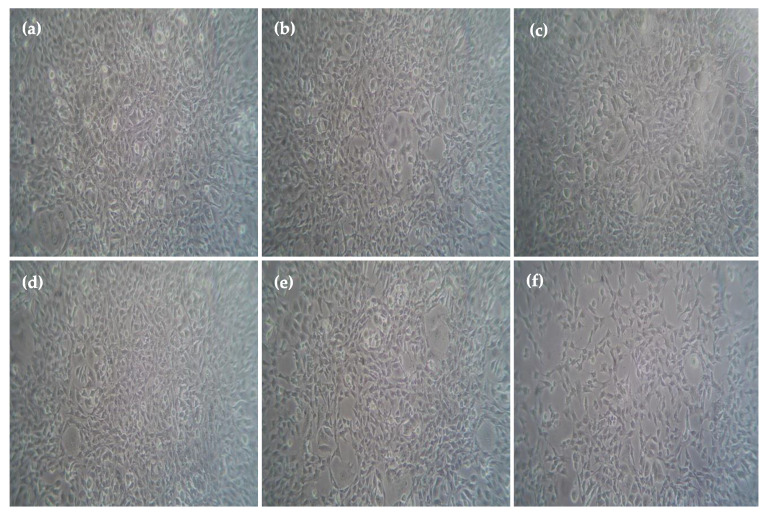
Anticancer activity of ethyl acetate extracts on MCF-7 (**a**) control, (**b**) 25 µg/mL, (**c**) 50 µg/mL, (**d**) 100 µg/mL, (**e**) 250 µg/mL, and (**f**) 500 µg/ mL.

**Figure 6:**
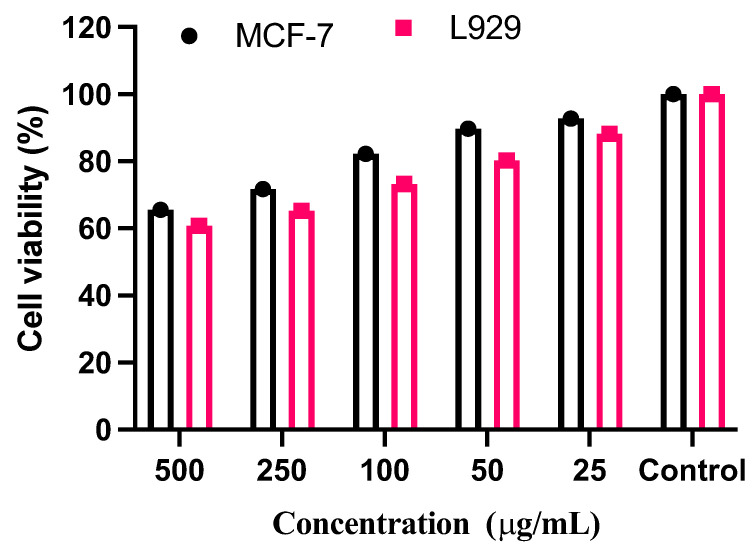
Anticancer activities of ethyl acetate extract from vegetable seed against MCF-7 and L929 cell line.

In vitro, the cytotoxic effect using ethyl acetate extract of vegetable seed on human breast cancer cell line (MCF-7) and normal fibroblast cell line (L929) was assessed by MTT assay. From Figure 4, Figure 5 and Figure 6, it is clear that the ethyl acetate extract showed potent cytotoxic effects against MCF-7 as well as L929 in a dose-dependent manner with the concentration of 500, 250, 100, 50, and 25 μg/mL showed 25.7 ± 0.3%, 88.3 ± 0.8%, 28.4 ± 0.3%, and 92.6 ± 0.6% cell viability, respectively. The five varied doses of the ethyl acetate seed extract were shown to be non-toxic to L929 cells. The results revealed a typical epithelial morphology in the control group with a high cell density. In contrast, shrinkage and rounding of cells, chromatin condensation, membrane blebbing, and the formation of apoptotic bodies with a decreased cell population were seen in cells treated with ethyl acetate extract and positive controls (Figure 4 and Figure 5). Morphological alterations might result from the caspase cascade being activated, which would cause the cleavage of the substrate poly (ADP-ribose) polymerase (PARP), which is necessary for the DNA repair pathway [42]. When a plant’s crude extract is taken in by a cell by endocytosis or micropinocytosis, ROS activates the apoptotic pathway, eventually causing cell death.

### 2.5. Molecular Docking Analysis

**Figure 7:**
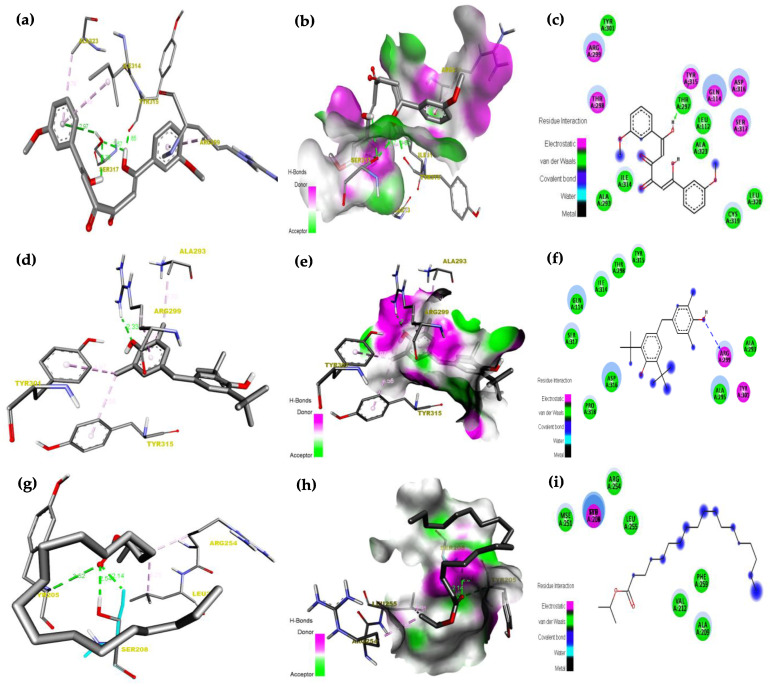
(**a**) Ligand–receptor hydrogen bond interactions of 3,4-Dihydroxy-1,6-bis-(3-methoxy-phenyl)-hexa-2,4-diene-1,6-dione. (**b**) Receptor side hydrogen bond interactions of3,4-Dihydroxy-1,6-bis-(3-methoxy-phenyl)-hexa-2,4-diene-1,6-dione. (**c**) 2D-ligand–receptor hydrogen bond interaction of 3,4-Dihydroxy-1,6-bis-(3-methoxy-phenyl)-hexa-2,4-diene-1,6-dione. (**d**) Ligand–receptor hydrogen bond interactions of Phenol,2,6-bis(1,1-dimethylethyl)-4-(4-hydroxy-3,5-dimethylphenyl)methyl). (**e**) Receptor side hydrogen bond interactions of Phenol,2,6-bis(1,1-dimethylethyl)-4-(4-hydroxy-3,5-dimethylphenyl)methyl). (**f**) 2D-ligand–receptor hydrogen bond interaction of Phenol,2,6-bis(1,1-dimethylethyl)-4-(4-hydroxy-3,5-dimethylphenyl)methyl). (**g**) Ligand–receptor hydrogen bond interactions of Isopropyl stearate. (**h**) Receptor side hydrogen bond interactions of Isopropyl stearate. (**i**) 2D-ligand–receptor hydrogen bond interaction of Isopropyl stearate.

A molecular docking method was used against receptor protein 1N0W. Its purpose is to calculate the binding modalities of GC-MS resultant ligands, namely, (1) 3,4-Dihydroxy-1,6-bis-(3-methoxy-phenyl)-hexa-2,4-diene-1,6-dione, (2) Phenol,2,6-bis(1,1-dimethyl ethyl)-4-(4-hydroxy-3,5-dimethyl phenyl)methyl)-, (3) Isopropyl stearate is acting a vital role in the structure and function of biological molecules, the ligand–receptor interactions were examined based on hydrogen bonding. The findings imply that all three ligands might effectively bind to the active site of 1N0W, as demonstrated in Table 3. The compound 3,4-Dihydroxy-1,6-bis-(3-methoxy-phenyl)-hexa-2,4-diene-1,6-dione was exhibited as a high binding energy value of −9.1(Kcal/mol), is due to the presence of low band gap energy of hydrogen bond (1.8Å), π-Donor Hydrogen Bond, and π-alkyl Hydrophobic bonds interaction between this ligand and 1N0W protein. The ligand–receptor hydrogen bond interactions, receptor side hydrogen bond interactions, and their two-dimensional (2D) pictures are shown in Figure 7. Besides, molecular docking investigation depicted that all three ligands could strongly occupy the active site of receptor protein 1N0W. It has been determined that such chemicals may target this region, preventing its function.

The ethyl acetate extracts from vegetable seeds reveal promising antimicrobial and antioxidant properties. Extracts from these plants showed significant antibacterial activity against Gram-positive bacteria, which is crucial given the rise in antibiotic resistance [14,43,44,45,46,47,48]. These extracts may serve as natural sources of antioxidants based on their antioxidant activity [31,40,49,50,51,52,53,54,55]. It was revealed that several bioactive compounds exist in this extract, including methyl oleate, isopropyl stearate, and 3,4-dihydroxy-1,6-bis-(3-methoxy-phenyl)-hexa-2,4-diene-1,6-dione [54,56,57,58]. There is evidence that such compounds contribute to the observed antimicrobial and antioxidant properties [31,50,52,53,54,55]. Furthermore, human breast cancer cells (MCF-7) were found to be cytotoxic in a dose-dependent manner, while normal fibroblast cells (L929) were not affected. Apoptotic pathways may be involved in this cytotoxicity. Furthermore, molecular docking analysis confirmed their ability to bind to the 1N0W receptor protein, indicating their potential therapeutic utility. According to previous studies, plant seed extracts are important for preventing antibiotic resistance and developing natural antioxidants [35,59]. Research in this area could investigate the individual contributions of different compounds to these biological activities and how they might be used therapeutically in the future.

## 3. Materials and Methods

### 3.1. Seed Material Collection

Fresh, disease-free vegetable seeds (synergistic) were procured from the botanical park in Chennai, India. The complete seed types were combined, sorted, cleaned, and air-dried at room temperature for 8–10 days. The vegetable seed types were processed into fine powder. When powdered samples were needed for the extraction procedure, they were collected and kept in airtight containers shielded from heat and sunlight.

### 3.2. Extract Preparation

Vegetable seeds (300 g) *Cucurbita maximas, Hibiscus cannabinus, Cyamopsis tetragonoloba, Phaseolus vulgaris,* and *Solanum melongena* (synergistic) that had been ground up were mixed consecutively for 70–72 h in a rotator shaker operating at 130 rpm with (1:3 ratio) 900 mL of organic solvents, such as hexane, ethyl acetate, and methanol. Once dry and solvent-free, the filtrates were further concentrated at 40 °C in a rotating evaporator. To conduct additional in vitro studies, the resulting extracts were stored at 4 °C [60].

### 3.3. Soxhlet Extraction

In accordance with the methodology described elsewhere [61], the extract preparation was evaluated. Approximately 900 mL of solvents, including hexane, ethyl acetate, and methanol, were employed for the extract of powdered (synergistic) vegetable seed types weighing 300 g. The extract was filtered in a sterile environment using a Seitz filter and Whatman filter paper, and the extracts were then produced in powder form using a lyophilizer set at a temperature of −80 °C. The prepared extracts were subsequently stored at 4 °C for further analysis.

### 3.4. In Vitro Antibacterial Assessment

For the antimicrobial assay, Gram-positive bacteria of *S. epidermis* (MTTC 3615) and *M. luteus* (MTCC 106), as well as Gram-negative bacteria *E. aerogens* (MTCC 111) and *P. vulgaris* (MTCC 1771) were used in this study. The Gram-positive and Gram-negative bacterial cultures were grown separately on Mueller-Hinton broth (pH 7.0). The cultures were incubated for 24 h at 37 °C on a rotating shaker. After incubation, the bacterial cultures were adjusted to a concentration of 1.5 × 10^8^ CFU/mL and were swabbed onto sterile Mueller–Hinton agar plates. Vegetable seed extracts in three different solvents (20 mL each), hexane, ethyl acetate, and methanol, were applied to sterile discs (6 mm) and left to soak for 10 to 15 min. The discs were aseptically transferred to the plates seeded with the appropriate pathogens and incubated at 37 °C for 24 h using sterile forceps. Following the 24 h incubation period, the various solvent extracts of vegetable seeds were assessed for their ability to generate a zone of inhibition (measured in mm) against the indicator pathogenic bacteria. Streptomycin and the respective solvent-soaked discs were used as positive and negative controls, respectively. The tests were performed in triplicate to ensure the reliability and accuracy of the results.

### 3.5. DPPH Radical Scavenging Assay

The Sayah et al. [55] technique was used to assess the DPPH radical scavenging capacity of the ethyl acetate seed extract. At 517 nm, the absorbance was measured in comparison to a blank. The equation below was used to estimate the DPPH scavenging activity, and the IC50 value was calculated. DPPH scavenging capacity (%) = [(A sample − A blank)/A control] × 100.

### 3.6. Hydrogen Peroxide (H_2_O_2_) Scavenging Activity

According to the methods of Ruch et al. [62], hydrogen peroxide scavenging characteristics of ethyl acetate extracts of seed extract at various concentrations (200–1000 µg/mL) used the following equation to calculate: Hydrogen peroxide scavenging (%) = [(A_0_ − A_1_)/A_0_] × 100. where A_0_ denotes the absorbance of the control, and A_1_ denotes the absorbance of the sample.

### 3.7. GC–MS Analysis

Following the methodology outlined by Venkatadri [63,64] using GC-MS (SHIMADZU QP2010, China) instrument, spectrum analysis was conducted to identify the phytochemical constituents present in the mid-polar solvent of ethyl acetate seed extract.

### 3.8. Anticancer Activities

The human breast cancer cell line (MCF-7) with passage number 15 and normal fibroblast cell line (L929) with passage number 9 were occupied from the National Centre for Cell Science in Pune, India.

### 3.9. Cytotoxicity

The monolayer cell culture was trypsinized using a medium containing 10% FBS, and the cell density was increased to 1.0 × 10^5^ cells/mL. MTT assay was performed to analyze the inhibitory concentration (IC_50_) of ethyl acetate seed extract. Both the cell lines were cultured in a microtiter plate for about 2 days to obtain 75% confluence. The medium was discarded, MTT was added to the culture, and incubated for 4 h at 37 °C. Then, the supernatant was removed, and about 50 µL of DMSO was loaded with the sample and incubated for 10 min. The absorbance was read at 570 nm using a microtiter plate reader, and the percentage viability was calculated [64]. The following formula was used to calculate the degree of inhibition, and the values for the test drug concentrations required to reduce cell growth by 50% (CTC50) for each line were obtained from the dose-response curves.

Percentage inhibition (%) [(OD_control_ − OD_sample_/OD_control_)] × 100

### 3.10. Molecular Docking Studies

To shed light on the examined ligands’ target-binding modes, molecular docking experiments were carried out (PDB code: 1N0W) [https://www.rcsb.org/structure/1N0W accessed between 12–20 March 2023 [39]. This protein controls the activity of RAD51, a recombinase enzyme, in the pathways for DNA repair by homologous recombination. It has been linked to a higher risk of developing breast cancer. Given the remarkable conservation of the RAD51 oligomerization motif, RecA-like recombinases have a common evolutionary origin for producing nucleoprotein filaments, reflected in the BRC repeat. Cancer-associated mutations disrupt the expected connection between the BRC repeat and RAD51, providing structural information about the processes underlying cancer susceptibility. We chose this 1N0W breast cancer-targeted protein as a result of docking investigations.

The molecular docking program Auto dock vina 4.2 was used to select the potential binding mode between the GC-MS resultant ligands, namely, (1) 3,4-Dihydroxy-1,6-bis-(3-methoxy-phenyl)-hexa-2,4-diene-1,6-dione, (2) Phenol,2,6-bis(1,1-dimethyl ethyl)-4-(4-hydroxy-3,5-dimethyl phenyl)methyl)-, (3) Isopropyl stearate and 1N0W receptor protein. The molecular docking program Auto dock vina4.2 was used to select the potential binding mode between the GC-MS resultant ligands, namely, (1) 3,4-Dihydroxy-1,6-bis-(3-methoxy-phenyl)-hexa-2,4-diene-1,6-dione, (2) Phenol,2,6-bis(1,1-dimethyl ethyl)-4-(4-hydroxy-3,5-dimethyl phenyl)methyl)-, (3) Isopropyl stearate and 1N0W receptor protein. ChemDraw 12.0 software was used to create the ligand structures. These 2D chemical structures undergo energy minimization before being translated to Protein Data Bank (PDB) format, which Auto Dock Vina4.2 then transforms into the PDBQT format structure. The Kollman unified atom charges and solvation parameters offered by Auto Dock Tools were used to add the appropriate hydrogen atoms to the 1N0W protein structure before docking. A-Chain, a protein from 1N0W, is chosen for docking investigations. To create ligand structures for docking, non-polar hydrogen (H) atoms, Gasteiger partial charges, and rotatable bonds were defined. A grid box with a maximum size of 92 × 94 × 100Å was created using Auto Grid. At the binding site for 1N0W, the grid box was allocated at the protein’s center using x, y, and z coordinates of 33.899, 26.022, and 1.196, respectively, with a grid spacing of 0.581 Å. Using Discovery studio visualizes, docked structures were shown. A two-dimensional (2D) graphic depicting the interaction between the protein and ligand was produced [42].

### 3.11. Statistical Analysis

Results from each experiment were given as Mean ± SD, with each experiment being performed in triplicate. The statistical study was conducted using Microsoft Excel 2007, and a straightforward linear regression curve was employed to determine the IC50 values. The *p*-value was only considered to be less than 0.05 for determining statistical significance.

## 4. Conclusions

The findings of this study highlight the significant antibacterial and antioxidant properties of the ethyl acetate extract derived from vegetable seeds. The extract exhibited cytotoxic effects against breast cancer cells (MCF-7) while demonstrating lower toxicity towards normal fibroblast cells (L-929). The synergistic extract of ethyl acetate seeds effectively inhibited cell growth and induced apoptosis in both MCF-7 and L-929 cells. These results indicate that vegetable seed extract possesses potential as a source of antibacterial, antioxidant, and anticancer agents. Further, in-silico molecular docking analysis 3,4-Dihydroxy-1,6-bis-(3-methoxy-phenyl)-hexa-2,4-diene-1,6-dione ligand, which has shown the best binding energy value of -9.1 kcal/mol to against target protein (PDB code: 1N0W) with Crystal Structure of Human and alkyl hydroperoxides by using reducing equivalents acquired from 3,4-Dihydroxy-1,6-bis-(3-methoxy-phenyl)-hexa-2,4-diene-1,6-dione. However, it is important to acknowledge certain limitations of this study. First, the study focused on a limited number of vegetable seed types, and further investigation involving a wider range of seed varieties would provide a more comprehensive understanding of their therapeutic potential. Additionally, the specific mechanisms underlying the observed antibacterial, antioxidant, and anticancer effects of the extract were not elucidated in this study. Future research should unravel the molecular pathways and signaling mechanisms involved. Despite these limitations, the results of this study pave the way for future research and development in the field of vegetable seed extracts. Further refinement and optimization of the extraction process are necessary to create a synergistic ethyl acetate extract from various vegetable seeds that can be effectively utilized as an active ingredient in pharmaceutical or nutraceutical formulations. Continued exploration of vegetable seed extracts holds promise for the development of novel therapeutic agents with enhanced efficacy and minimal adverse effects.

## Figures and Tables

**Table 1:** Antibacterial activity of vegetable seed extracts against Gram-positive and Gram-negative bacteria.

Tested Microorganisms	Streptomycin (mm)	Zone of Inhibition (mm) at Concentration in (μg/mL)		
		Hexane (mm)	Ethyl Acetate (mm)	Methanol (mm)
*Staphylococcus epidermis*	16.6 ± 0.5	8.6 ± 0.5	12.6 ± 0.5	8.3 ± 0.5
*Micrococcus luteus*	15.3 ± 0.5	7.6 ± 0.5	10.3 ± 0.5	8.6 ± 0.5
*Enterobacter aerogens*	15.3 ± 0.5	8.3 ± 0.5	11.5 ± 0.5	7.6 ±0.5
*Proteus vulgaris*	14.3 ± 0.5	7.6 ± 0.5	9.3 ± 0.5	8.6 ± 0.5

**Table 2:** Phyto-compounds analysis of ethyl acetate vegetable seed extracts present by GC MS.

Peak#	Molecular Formula	Retention Time	Identification
1	C_9_H_12_O	13.43	Phenol,2-Prophyl-
2	C_11_H_8_O_5_	14.17	Cumarin-3-carboxylic acid,7-methoxy
3	C_15_H_10_O_2_	15.17	Flavone
4	C_16_H_28_O	16.15	5-Cyclohexadecen-1-one
5	C_17_H_34_O_2_	17.12	Hexadecanoic acid, methyl ester
6	C_19_H_36_O_2_	18.85	Methyl oleate
7	C_21_H_42_O_2_	20.98	Isopropyl stearate
8	C_23_H_32_O_2_	22.82	Phenol,2,6-bis(1,1-dimethylethyl)-4-(4-hydroxy-3,5-dimethylphenyl)methyl)-
9	C_20_H_18_O_6_	24.3	3,4-Dihydroxy-1,6-bis-(3-methoxy-phenyl)-hexa-2,4-diene-1,6-dione

**Table 3:** The ligand–receptor residue interaction between Biscumarin ligand with 2JER protein.

S.NO	Pub Chem. Id	Ligand Name	Binding Energy (kcal/mol)	Residue Interaction	Type of Bond	Distance(Å)
1	1N0W	3,4-Dihydroxy-1,6-bis-(3-methoxy-phenyl)-hexa-2,4-diene-1,6-dione	−9.2	: UNK0: H-A: TYR315: O	Hydrogen	1.8
				: UNK0: H-A: SER317: O	Hydrogen	2.5
				: UNK0: H-A: SER317 O	Hydrogen	2.7
				A: SER317: H-:UNK0	π-Donor Hydrogen Bond	2.9
				: UNK0-A: ARG299	π-alkyl Hydrophobic	4.3
				: UNK0-A: ILE314	π-alkyl Hydrophobic	4.8
				: UNK0-A: ALA323	π-alkyl Hydrophobic	4.7
2	1N0W	Phenol,2,6-bis(1,1-dimethylethyl)-4-(4-hydroxy-3,5-dimethylphenyl)methyl)-	−6.8	A: ARG299: H-:UNK0: O	Hydrogen Bond	2.3
				A: ALA293-:UNK0: C	alkyl Hydrophobic	3.6
				: UNK0-A: ARG299	π-alkyl Hydrophobic	3.9
				A: TYR301-:UNK0: C	π-alkyl Hydrophobic	4.9
				A: TYR315-:UNK0: C	π-alkyl Hydrophobic	4.5
3.	1N0W	Isopropyl stearate	−4.0	A: SER208: H-:UNK0: O	Hydrogen Bond	2.1
				A: SER208: H-:UNK0: O	Hydrogen Bond	2.5
				A: TYR205: C-:UNK0: O	Hydrogen Bond	3.5
				: UNK0:C-A: ARG254	alkyl Hydrophobic	3.6
				: UNK0-C-A: LEU255	alkyl Hydrophobic	4.2

## Data Availability

The data created is/are cited in the manuscript.
